# Dynamic transcriptomic responses to soybean cyst nematode infection in soybean genotypes with contrasting resistance profiles

**DOI:** 10.3389/fpls.2025.1618387

**Published:** 2025-08-21

**Authors:** Nour Nissan, Nathalie Puchacz, Julia C. Hooker, Dave T. Ste-Croix, Gerardo Zapata, François Lefebvre, Martin Charette, Ashkan Golshani, Elroy Cober, Benjamin Mimee, Bahram Samanfar

**Affiliations:** ^1^ Ottawa Research and Development Centre, Agriculture and Agri-Food Canada, Ottawa, ON, Canada; ^2^ Department of Biology, Ottawa Institute of Systems Biology, Carleton University, Ottawa, ON, Canada; ^3^ Saint-Jean-sur-Richelieu Research and Development Centre, Agriculture and Agri-Food Canada, Saint-Jean-sur-Richelieu, QC, Canada; ^4^ Canadian Centre for Computational Genomics, Montréal, QC, Canada

**Keywords:** soybean cyst nematode, SCN, soybean, RNA-seq, differential expression, SCN resistance

## Abstract

**Introduction:**

Soybean cyst nematode populations are rapidly evolving to overcome the limited genetic resistance currently employed in commercial soybean varieties, threatening the future of crop production. To mitigate that, it is crucial to identify novel sources of resistance. Soybean lines PI 561310 and PI 567295 were previously found to exhibit partial SCN resistance despite lacking resistant alleles at *rhg1* and *Rhg4*.

**Methods:**

In an attempt to elucidate their resistance mechanisms, PI 561310 and PI 567295 alongside susceptible Essex and resistant Peking and PI 88788 lines were infected with SCN HG type 0. Control and infected root tissues were collected at 5 and 10 day post-infection for RNA sequencing and comparative transcriptomic response analyses were performed.

**Results and discussion:**

The resistance mechanism employed by PI 561310 appears to be driven by the expression of *PAL*, which positively regulates downstream lignin, flavonoid and salicylic acid biosynthesis pathways. PI 567295 displayed a unique and divergent transcriptomic response characterized predominantly by gene downregulation, rather than the canonical upregulation observed during defense activation; an atypical response suggestive that PI 567295 employs a distinct mechanism not previously described in the literature. This study explores the diversity of defense strategies and provides valuable insight into novel genetic origins of SCN resistance as we navigate the landscape of evolving SCN parasitism.

## Introduction

Cultivated soybean (*Glycine max* (L.) Merr.) is an economically important crop that is widely utilized in food, feed, and biofuel production. The growth of soybean crops play a crucial role in sustainable agricultural practices due to its symbiotic relationship with nitrogen-fixing bacteria (*Bradyrhizobia japonicum*), which helps minimize the requirements for nitrogen fertilizers (Boerema et al., 2016). However, soybean yields are threatened by various biotic stressors, including a major pathogen known as *Heterodera glycines Ichinohe*, or more commonly as soybean cyst nematode (SCN) (Bradley et al., 2021). First identified in Japan in 1915 and in the United States in 1954, the nematode is now distributed worldwide (Arjoune et al., 2022; Winstead et al., 1955). Soybean cyst nematode poses a significant threat to soybean crops, with the annual economic impact estimated at around $1.5 billion USD in the United States and $30 to 50 million CAD in Ontario (Bandara et al., 2020; Bradley et al., 2021).

Soybean cyst nematode are parasites that infect the roots of the soybean plant through the formation of specialized feeding sites called syncytia, which divert nutrients from the plant for its own reproductive success (Gheysen and Mitchum, 2011). The parasitic second-staged juveniles will pierce and enter the roots, establishing their syncytia near the vascular bundles where they will feed for up to four weeks until they reach the adult stage (Hu et al., 2018). Soybean cyst nematode infection can cause more than 30% yield loss without the manifestation of additional outward symptoms and its eradication once present in fields is dismal. The application of nematicides is costly and environmentally harmful, serving primarily as a short-term solution. Rotation of non-host crops such as oats, corn, sorghum, wheat and alfalfa can be effective, but aren’t always profitable and practical for farmers. Moreover, the cysts of SCN can survive for years in fields that have been absent of soybean crops, making complete elimination of the pathogen impossible through crop rotation alone (Arjoune et al., 2022; Hu et al., 2018).

Currently, there are three genetic resistance sources commercially used for SCN resistance in soybean, PI 437654 (Hartwig or CystX), PI 54802 (Peking) and PI 88788. Two major-effect resistance loci are *rhg1* (*Resistance to Heterodera glycines 1*) and *Rhg4*. The *rhg1* locus is found on chromosome 18 and consists of a 31.2 kb four-gene block segment that codes for three different proteins that function in resistance to SCN infection (Cook et al., 2012). The first gene encodes for an amino acid transporter (AAT) (*GmAAT*; Glyma.18G022400), the second gene encodes for an α-SNAP protein [soluble NSF (N-ethylmaleimide-sensitive factor) attachment protein] (*GmSNAP18*; Glyma.18G022500), and the third gene encodes for a wound-inducible domain protein (WI12) (*GmWI12*; Glyma.18G022700) (Cook et al., 2012; Liu et al., 2017). Three haplotypes in reference to this locus had originally been documented based on the observed copy-number variation: *rhg1-a* type, *rhg1-b* type and *rhg1-c* type. *Rhg1-a* type (also identified as the Peking-type resistance or “low copy number”) refers to the presence of 2–3 tandem copies of *rhg1*, *rhg1-b* type (also identified as the PI 88788-type resistance or “high copy number”) refers to the presence of 7–10 tandem copies of *rhg1*, and *rhg1-c* type which refers to the presence of only a single copy of *rhg1* and has been found in susceptible cultivars, such as Williams 82 and Essex (Cook et al., 2012). In addition to copy-number variations, genetic structural variations have been observed in the genes encoded by *rhg1*, subsequently producing varying alleles of each gene. In order to be classified as the *rhg1-a* haplotype, *rhg-1* must not only be present in low-copy numbers but also carry a retrotransposon in the GmSNAP18 gene, which causes C-terminal polymorphisms at a conserved functional site and produces the distinctive resistance-type α-SNAP proteins, which is not present in the *rhg1-b* or *rhg1-c* haplotypes (Bayless et al., 2019; Cook et al., 2014). Transcriptional analysis of *rhg-1* resistance genes have found that transcript level abundance scales proportionately with *rhg1* copy number and results in elevated constitutive transcription under non-infected conditions (Cook et al., 2014).

The *Rhg4* locus is one of the resistance QTLs derived from Peking and PI 437654, which is found on chromosome 8 (Liu et al., 2012). Similar to the *rhg1* locus, the *Rhg4* locus consists of a 35.7 kb tandem repeat segment of a three-gene block composed of Glyma.08G108800, Glyma.08G108900, and Glyma.08G109000. Two haplotypes in reference to this locus have been documented based on observed copy-number variation; *rhg4-a* type, which is observed to have 1-4.3 copies and is identified in Peking-type resistance, and *rhg4-b* type which is observed to have a single copy and is identified as the wild-type (Patil et al., 2019). The gene responsible for conferring resistance to SCN has been identified to encode a polymorphic serine hydroxymethyltransferase (SHMT) (*GmSHMT08;* Glyma.08G108900), whose promoter sequence differs between resistant and susceptible lines (Kandoth et al., 2017; Liu et al., 2012; Patil et al., 2019). Lines harboring Peking-type resistance (*rhg1-a*/“low copy-number”) only exhibit “full-strength” resistance to SCN infection when *rhg1-a* is present in an epistatic interaction with the specific allele of SHMT at *Rhg4* (Kandoth et al., 2017; Liu et al., 2012; Patil et al., 2019). Lines harboring PI 88788-type resistance (*rhg1-b*/“high copy-number”) do not benefit from the presence of any specific *Rhg4* alleles, but rather appear to rely on high-copy numbers, at least 5.6 copies, of *rhg1* (Patil et al., 2019).

Unfortunately, due to the persistent use of these resistance genes SCN populations are evolving to overcome these resistance loci, resulting in diminished efficacy (Arjoune et al., 2022). The continued reliance on *rhg1* and *Rhg4* gene-based resistance as the sole sources of resistance in breeding programs and in the field is not a sustainable strategy and necessitates the identification of novel resistant sources and genes to maintain successful soybean production worldwide. Luckily, there have been resistant soybean lines found that do not carry the resistant alleles of *rhg1* or *Rhg4*, as well QTLs identified to play a role in resistance against SCN (Nissan et al., 2022; Tran et al., 2019).

RNA-sequencing is a powerful tool for the qualitative and quantitative assessment of gene expression. Analysis of differential gene expression enables the discernment of crucial information pertaining to transcript abundance and nucleotide sequence variation, providing researchers with insight into underlying molecular processes (Griffith et al., 2015). Sequencing the transcriptomes of control and infected plants allows us to explore the molecular defense mechanisms triggered by specific pathogens. In the context of SCN infection in soybean, these approaches can be instrumental in identifying genes that contribute to resistance, particularly in newly recognized SCN-resistant lines. In this study, we analyzed the transcriptomes of five soybean lines with varying degrees of resistance to infection with SCN HG type 0; the susceptible line Essex, two resistant lines carrying *rhg*-based resistance mechanisms: Peking and PI 88788, and two lines that have demonstrated partial resistance through mechanisms that were identified as not *rhg*-based: PI 561310 and PI 567295 (Tran et al., 2019). Using RNA-sequencing, we analyzed the root tissues from each line under both non-infected and infected conditions at 5- and 10-day post-infection (DPI) to capture temporal changes in defense responses, and validated our data using RT-qPCR.

## Materials and methods

### SCN population and soybean lines

The five soybean lines used in this study: Essex, Peking, PI 88788, PI 561310 and PI 567295 were obtained from the USDA Soybean Germplasm Collection. The soybean cyst nematode population used in this study was validated as HG type 0 (race 3) by evaluating the female index (FI) on each indicator lines at 34-DPI as described in (Niblack et al., 2002), using Essex as the susceptible control. PI 561310 and PI 567295 were additionally included to determine their resistance level to the SCN population used in this study. PI 88788 and Peking are known to possess *rhg1-* and *Rhg4-*based resistance mechanisms, while PI 561310 and PI 567295 have been previously identified as partially resistant without possessing *rhg1-* and *Rhg4-*based resistance haplotypes (Tran et al., 2019).

### Experimental design and sample collection

A greenhouse experiment was carried out at Agriculture and Agri-Food Canada, Saint-Jean-sur-Richelieu Research and Development Centre, QC, Canada. The experimental design was comprised of two treatment conditions: non-infected controls (CTR) and SCN-infected (INF) plants, for each of the five soybean lines: Essex, Peking, PI 88788, PI 561310 and PI 567295 in triplicates for a total of 60 samples. Seeds from each of the five soybean lines were germinated in moist Pro-Mix for five days in a growth cabinet held at 28˚C, with 50% relative humidity (RH) and a 12h:12h photoperiod until they reached the VE stage. Seedlings were then transferred to individual SC10U Ray Leach “Super Cell” UV cone-tainers (Stuewe and Sons Inc. Corvallis, OR, USA) filled with a moist mixture of 50:50% beach and masonry sand, then inoculated with a suspension of 2,000 SCN eggs from the SCN population of HG Type 0 (race 3). Two series of buckets were prepared to assess gene expression at 5 days post-infection and 10 days post-infection. Buckets were maintained at 28˚C in a temperature-controlled water table to a 50% RH and a 16h photoperiod. Roots were washed free of sand and flash-frozen in liquid nitrogen and stored at -80°C prior to downstream RNA sequencing.

### RNA extraction, library construction and RNA sequencing

Frozen root samples were ground in liquid nitrogen with mortar and pestle followed by a total RNA extraction using the RNeasy Plant Mini kit (Qiagen, Venlo, Netherlands) according to the manufacturer’s instructions. RNA integrity was analyzed using a 2100 Bioanalyzer (Agilent, Santa Clara, USA). cDNA libraries were prepared and paired-end (2 x 100bp) sequenced using the Illumina NovaSeq 6000 PE100-25M reads platform (Illumina, San Diego, USA) at Génome Québec in Montréal, QC, Canada.

### Sequence alignment, mapping, read counts and differential expression analysis

To ensure accurate gene expression data, only samples with RIN value > 8 were kept. We used GenPipes, the main in-house framework of the Canadian Center for Computational Genomics (C3G), to perform all major processing steps (Bourgey et al., 2019). Briefly, adaptor sequences and low-quality score containing bases (Phred score < 30) were trimmed from reads using Trimmomatic (Bolger et al., 2014). The resulting reads were then aligned to the genome (Glycine max v2.1, GCF_000004515.4), using the splice-aware aligner STAR (Dobin et al., 2013). Read counts were extracted from bam files using HTSeq (Anders et al., 2015). The R package DESeq2 was used to identify differences in expression levels between the groups using negative Binomial GLM fitting and Wald statistics: nbinomWaldTest (Love et al., 2014). Similarly “ashr” was used to shrink log_2_ fold changes in gene expression data (Stephens, 2017). Genes were considered differentially expressed at an adjusted p-value ≤ 0.01 and log_2_fold change difference in expression ≥ 1 for this analysis.

### Functional annotation, enrichment and pathway analyses

Venn diagrams for inter-line transcriptomic response analyses were generated using: https://bioinformatics.psb.ugent.be/webtools/Venn/. The gene ontology biological process (GO: BP) data for each of the genes of interest identified in these contrasts was extracted from SoyBase (https://soybase.org) and Arabidopsis homologs were extracted from the TAIR10 database (https://www.arabidopsis.org). KEGG pathway enrichment was done using the Mapper search function from the Kyoto Encyclopedia of Genes and Genomes (KEGG) release v106.0 (https://www.kegg.jp/), with Glycine max (gmx) as the organism’s identifier specified.

### RT-qPCR for validation of differentially expressed genes

To validate the RNA-sequencing results, total RNA was re-extracted from root samples using the procedures mentioned earlier. Total RNA was then reverse transcribed into cDNA using the iScript synthesis kit according to the manufacturer’s protocol (BioRad Laboratories, Hercules, CA, USA). Genes found similarly expressed in both PI 561310 and PI 567295 at either 5-DPI or 10-DPI were selected and underwent RT-qPCR using the SsoAdvanced Universal SYBR Green PCR SuperMix (BioRad Laboratories, Hercules, CA, USA). The housekeeping gene Elongation factor 1b (Glyma.02G276600) was used for normalization purposes and samples were analyzed in triplicates. Fold change values were calculated using the pfaffl method (Pfaffl, 2001). Primer sequences used to amplify genes are listed in **Supplementary Table 2**.

## Results

### Evaluation of greenhouse SCN bio-assay

The resistance levels of the soybean lines used in this study; Peking, PI 88788, PI 561310 and PI 567295 to SCN HG type 0 were assessed as described in (Niblack et al., 2002), using Essex as the susceptible control. Essex was confirmed as susceptible to infection, with Peking and PI 88788 resistant to infection through known *rhg*-based resistance mechanisms; all three resistance ratings validated through our FI analysis (**Table 1**). Two lines included in this study: PI 561310 and PI 567295 were previously found to exhibit partial resistance to SCN HG type 0 without possessing either resistant *rhg* alleles, both identified as moderately resistant (Tran et al., 2019). Our FI analysis (**Table 1**) confirmed PI 561310 as moderately resistant, and classified PI 567295 as moderately susceptible. The rating differences between PI 561310 and PI 567295 are due to the cutoff for moderately resistant FI < 30; the FI values of PI 561310 and PI 567295 are quite close.

**Table 1:** Summary of FI results of tested soybean lines against SCN HG type 0 and their subsequent resistance ratings.

Soybean cultivar	Female index (%)	Rating
Essex*	100.00 ± 6.59	Susceptible
Peking	2.20 ± 0.05	Resistant
PI 88788	1.87 ± 0.17	Resistant
PI 561310	29.10 ± 0.84	Moderately Resistant
PI 567295	33.80 ± 1.27	Moderately Susceptible

*The number of females developing on the susceptible control Essex varied between 414 and 515.

### Evaluation of RNA-sequencing sample variability

**Figure 1:**
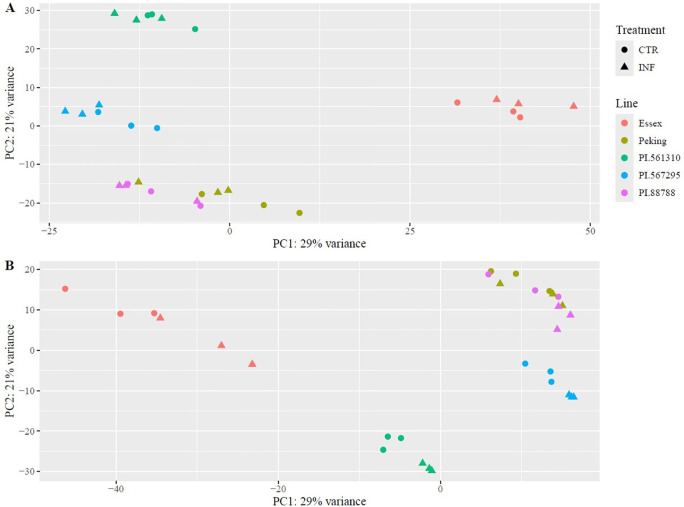
PCA for normalized RNA-sequencing expression data for infected and non-infected soybean line samples at **(A)** 5-DPI and **(B)** 10-DPI. Data is plotted with sample treatment represented by shapes, with non-infected treatment samples (CTR) represented as a circle and infected treatment samples (INF) represented as a triangle, with each line distinguished by color.

There was a total of 4,284,400,682 raw reads over all 60 RNA-sequencing datasets (five lines, two treatment conditions, two timepoints and three replicates per line). The trimmed reads were aligned to the *Glycine max* Williams 82 reference genome (*Glycine max* v2.1), with an 89 – 95% alignment efficiency of the transcriptome to the reference in the 60 samples (**Supplementary Table 1**). The expression profiles of the resistant lines PI 88788 and Peking clustered together and were distinct from the susceptible line Essex on the two main axes of the principal component analysis (**Figure 1**). The partially resistant lines PI 561310 and PI 567295 closely resembled PI 88788 and Peking on one principal axis, while on the other axis, PI 567295 showed a profile similar to Essex, and PI 561310 displayed a profile distinct from all other lines. Although the expression profiles of non-infected (CTR) and infected (INF) treatment replicates within individual lines clustered together, due to the significant differences between plant genotypes, they also exhibited differences, when looked individually, that became more pronounced over time.

### Intra-line transcriptomic response analysis

**Figure 2:**
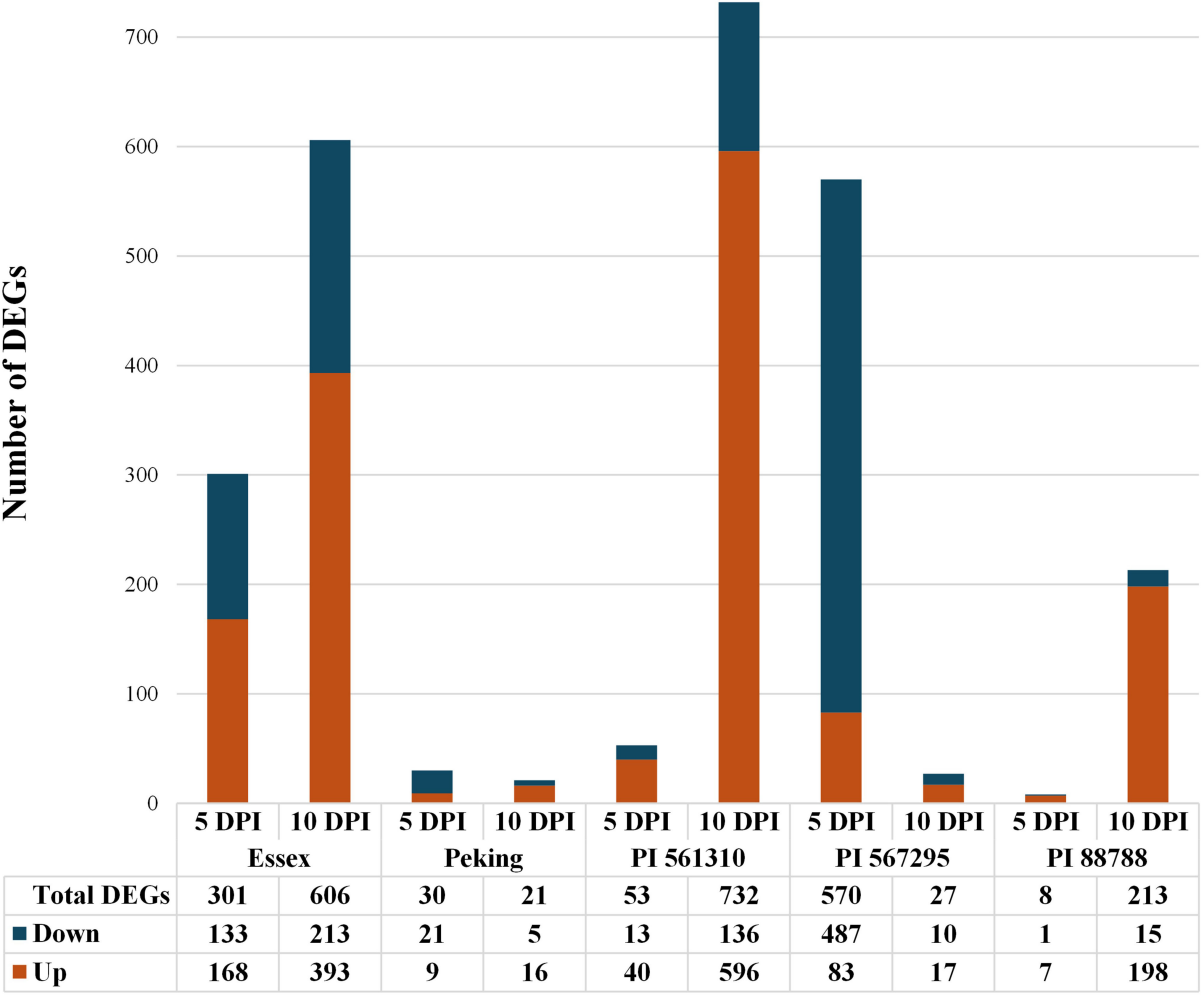
Stacked bar chart depicting the number of differentially expressed genes (DEGs) identified from each of the five soybean lines at 5-DPI and 10-DPI, in infected treatments compared to non-infected treatments. DEGs are divided into upregulated genes and downregulated gene fractions, with upregulated genes represented in orange and downregulated genes represented in blue.

A comparative analysis of the transcriptomic response of each soybean line to SCN infection was conducted by identifying differentially expressed genes (DEGs) in infected samples compared to non-infected samples within each line at 5-DPI and 10-DPI, respectively. Differentially expressed genes were considered significant at an adjusted p-value ≤ 0.01 and log_2_ fold change difference in expression ≥ 1 for this analysis. **Figure 2** shows the number of identified DEGs, with the breakdown of upregulated and downregulated gene fractions, for each line at 5-DPI and 10-DPI. Subsequent functional enrichment analyses of Gene Ontology (GO) terms considered all the genes which were differentially expressed.

#### Essex

At 5-DPI, Essex had 301 DEGs (168 upregulated, 133 downregulated), with enriched categories related to heat response, carbohydrate metabolism, and protein phosphorylation. By 10-DPI, the number of DEGs doubled to 606 (393 upregulated, 213 downregulated), with additional enrichments in defense response and oxidation-reduction processes. KEGG pathway analysis revealed involvement in processes such as plant hormone signal transduction, MAPK signaling, and starch and sucrose metabolism, with notable modules including acylglycerol degradation and triacylglycerol biosynthesis.

#### Peking

At 5-DPI, Peking exhibited only 30 DEGs (9 upregulated, 21 downregulated), decreasing to 21 DEGs at 10-DPI (16 upregulated, 5 downregulated). Key functional enrichments included responses to heat and regulation of transcription. KEGG pathway analysis highlighted pathways like the spliceosome and protein processing in the endoplasmic reticulum, with limited module involvement.

#### PI 88788

At 5-DPI, PI 88788 had the lowest number of DEGs (8 DEGs, 7 upregulated, 1 downregulated) but showed a significant increase to 213 DEGs at 10-DPI (198 upregulated, 15 downregulated). Enriched functions included protein phosphorylation and oxidative stress response. KEGG pathways such as phenylpropanoid biosynthesis and MAPK signaling were prominent, particularly at 10-DPI, where modules related to flavonoid and fatty acid biosynthesis were identified.

#### PI 561310 (moderately resistant)

**Figure 3:**
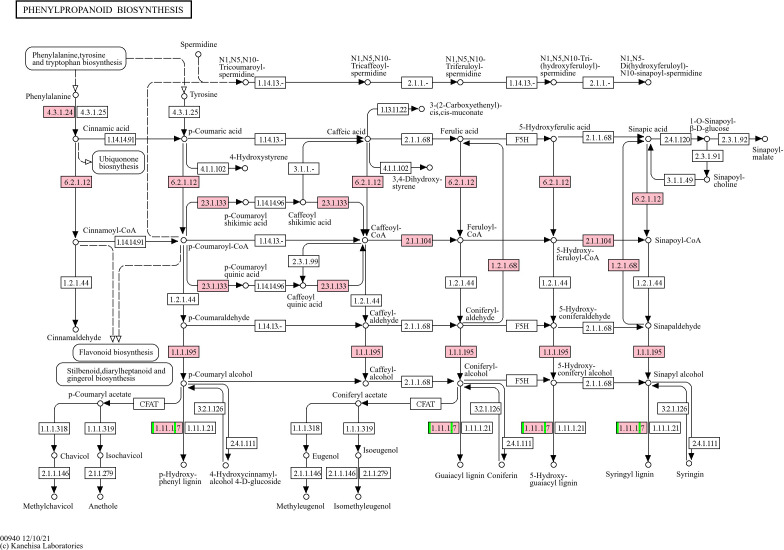
KEGG Phenylpropanoid Biosynthesis Pathway (gmx00940) showing the mapped DEGs from PI 561310 at 10-DPI. Pink boxes represent the presence of mapped upregulated genes, green boxes would represent the presence of mapped downregulated genes, and split-colored boxes represent the presence of both mapped upregulated and downregulated genes.

**Figure 4:**
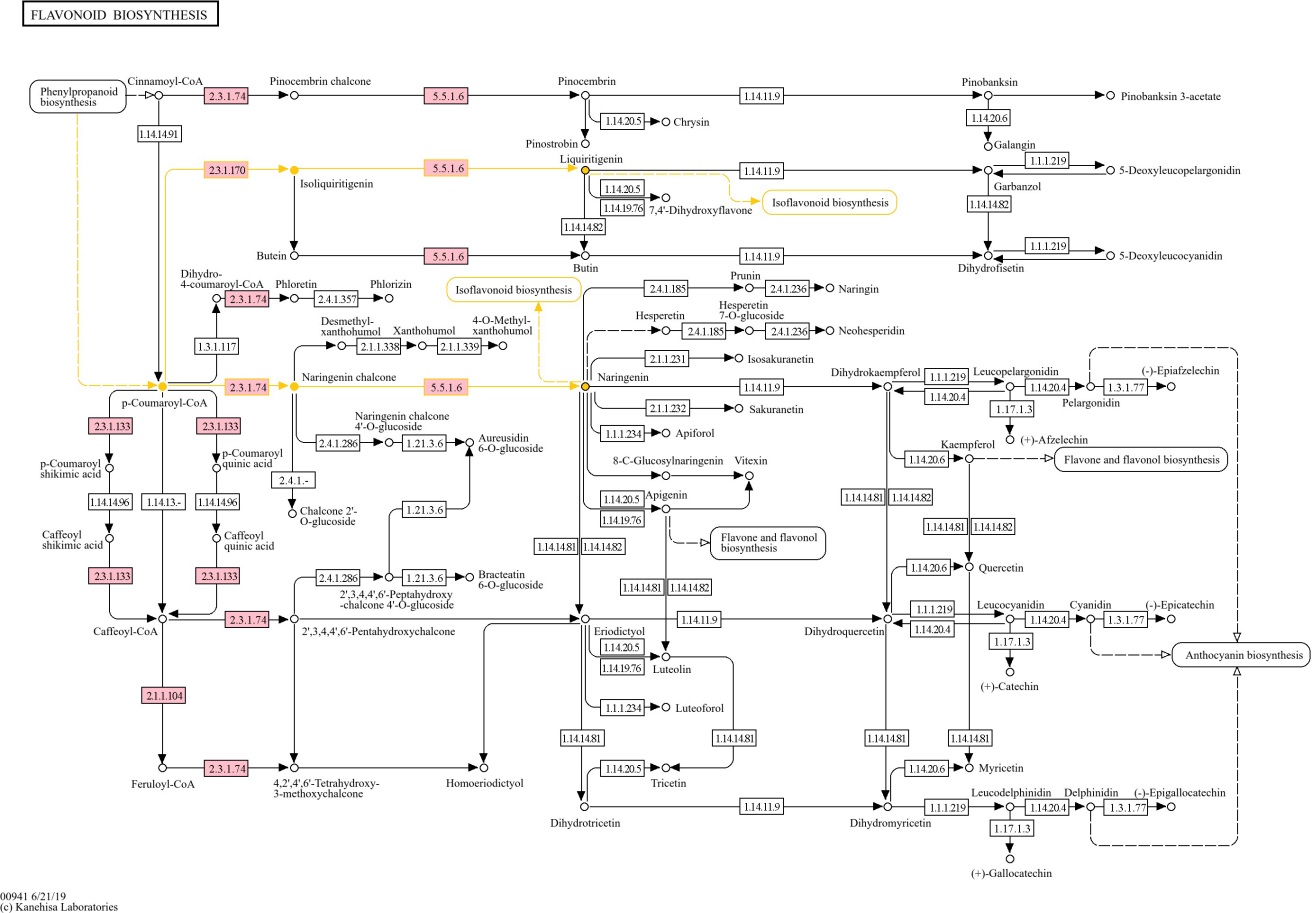
KEGG Flavonoid Biosynthesis Pathway (gmx00941) showing the mapped DEGs from PI 561310 at 10-DPI. Pink boxes represent the presence of mapped upregulated genes. Yellow highlighting specifies reaction flow in which phenylpropanoid biosynthesis product coumaroyl-CoA is used to produce the flavanones liquiritigenin and naringenin, which will then be used to produce key isoflavone compounds.

**Figure 5:**
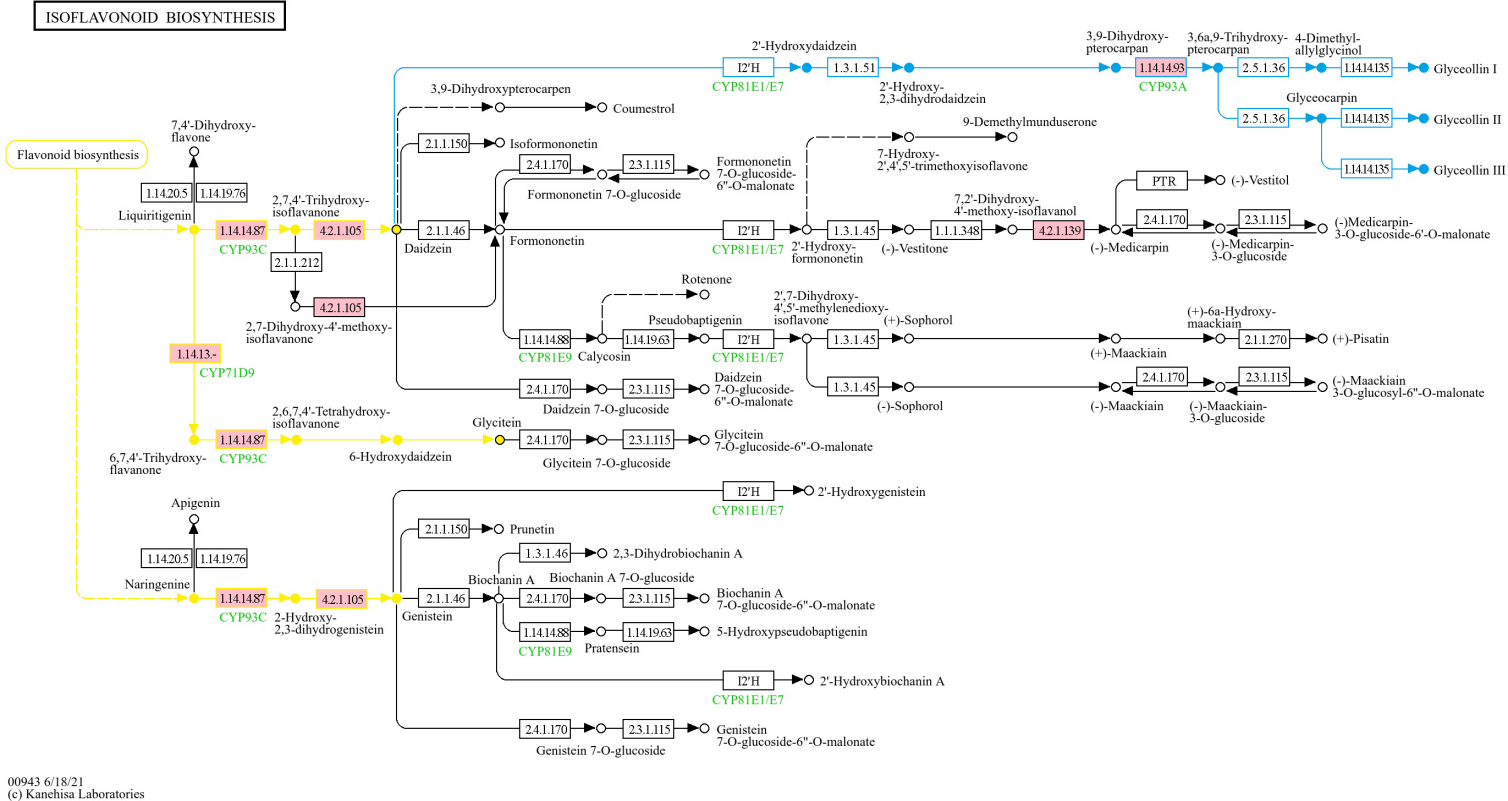
KEGG Isoflavonoid Biosynthesis Pathway (gmx00943) showing the mapped DEGs from PI 561310 at 10-DPI. Pink boxes represent the presence of mapped upregulated genes. Yellow highlighting specifies reaction flow in which flavonoid biosynthesis pathway products liquiritigenin and naringenin is used to produce isoflavone compounds glycitein, genistein and daidzein. Blue highlighting specifies reaction flow where daidzein is used to produce glyceollins.

At 5-DPI, PI 561310 had 53 DEGs (40 upregulated, 13 downregulated), with enrichments in carbohydrate metabolism and oxidation-reduction processes. The number of DEGs increased to 732 at 10-DPI (596 upregulated, 136 downregulated), showing strong enrichment in biotic stimulus response and metal ion transport. KEGG analysis highlighted pathways like phenylpropanoid biosynthesis, MAPK signaling, and starch metabolism, with modules involving flavonoid and isoflavonoid biosynthesis particularly enriched (**Figures 3**–**5**, **Table 2**).

**Table 2:** List showing the number of mapped DEGs from PI 561310 at 10-DPI into various KEGG modules.

KEGG module	KEGG module pathway	Number of genes
gmx_M00137	Flavanone biosynthesis, phenylalanine => naringenin	7
gmx_M00039	Monolignol biosynthesis, phenylalanine/tyrosine => monolignol	7
gmx_M00940	Flavanone biosynthesis, p-coumaroyl-CoA => liquiritigenin	5
gmx_M00941	Isoflavone biosynthesis, liquiritigenin/naringenin => daidzein/genistein	3
gmx_M00001	Glycolysis (Embden-Meyerhof pathway), glucose => pyruvate	3
gmx_M00942	Pterocarpan biosynthesis, daidzein => medicarpin	3
gmx_M00022	Shikimate pathway, phosphoenolpyruvate + erythrose-4P => chorismate	2
gmx_M00910	Phenylalanine biosynthesis, chorismate => arogenate => phenylalanine	2
gmx_M00002	Glycolysis, core module involving three-carbon compounds	2
gmx_M00370	Glucosinolate biosynthesis, tryptophan => glucobrassicin	2
gmx_M00171	C4-dicarboxylic acid cycle, NAD - malic enzyme type	2
gmx_M00113	Jasmonic acid biosynthesis	2
gmx_M00034	Methionine salvage pathway	2
gmx_M00003	Gluconeogenesis, oxaloacetate => fructose-6P	2
gmx_M00119	Pantothenate biosynthesis, valine/L-aspartate => pantothenate	2
gmx_M00570	Isoleucine biosynthesis, threonine => 2-oxobutanoate => isoleucine	1
gmx_M00932	Phylloquinone biosynthesis, chorismate (+ phytyl-PP) => phylloquinol	1
gmx_M00036	Leucine degradation, leucine => acetoacetate + acetyl-CoA	1
gmx_M00169	CAM (Crassulacean acid metabolism), light	1
gmx_M00044	Tyrosine degradation, tyrosine => homogentisate	1
gmx_M00040	Tyrosine biosynthesis, chorismate => arogenate => tyrosine	1
gmx_M00172	C4-dicarboxylic acid cycle, NADP - malic enzyme type	1
gmx_M00914	Coenzyme A biosynthesis, archaea, 2-oxoisovalerate => 4-phosphopantoate => CoA	1
gmx_M00849	C5 isoprenoid biosynthesis, mevalonate pathway, archaea	1
gmx_M00913	Pantothenate biosynthesis, 2-oxoisovalerate/spermine => pantothenate	1
gmx_M00133	Polyamine biosynthesis, arginine => agmatine => putrescine => spermidine	1
gmx_M00371	Castasterone biosynthesis, campesterol => castasterone	1
gmx_M00873	Fatty acid biosynthesis in mitochondria, animals	1
gmx_M00368	Ethylene biosynthesis, methionine => ethylene	1
gmx_M00019	Valine/isoleucine biosynthesis, pyruvate => valine/2-oxobutanoate => isoleucine	1
gmx_M00096	C5 isoprenoid biosynthesis, non-mevalonate pathway	1
gmx_M00372	Abscisic acid biosynthesis, beta-carotene => abscisic acid	1
gmx_M00345	Formaldehyde assimilation, ribulose monophosphate pathway	1
gmx_M00023	Tryptophan biosynthesis, chorismate => tryptophan	1
gmx_M00116	Menaquinone biosynthesis, chorismate (+ polyprenyl-PP) => menaquinol	1
gmx_M00112	Tocopherol/tocotorienol biosynthesis, homogentisate + phytyl/geranylgeranyl-PP => tocopherol/tocotorienol	1
gmx_M00095	C5 isoprenoid biosynthesis, mevalonate pathway	1
gmx_M00532	Photorespiration	1
gmx_M00527	Lysine biosynthesis, DAP aminotransferase pathway, aspartate => lysine	1
gmx_M00024	Phenylalanine biosynthesis, chorismate => phenylpyruvate => phenylalanine	1
gmx_M00173	Reductive citrate cycle (Arnon-Buchanan cycle)	1

Information includes KEGG module code, KEGG module pathway with specified reaction process, and the number of DEGs mapped into the modules.

Several upregulated genes in PI 561310 at 10-DPI were found to map into KEGG pathways and modules (**Table 2**) that pertain to the flavonoid biosynthesis process, illustrating the flow of the reaction cascade: 1) Eight genes were found to map into the flavonoid biosynthesis pathway gmx00941 (**Figure 4**), along with seven and five genes mapping into the flavanone biosynthesis module gmx_M00137 and flavanone biosynthesis module gmx_M00940, respectively. 2) Eight genes were found to map into the isoflavonoid biosynthesis pathway gmx00943 (**Figure 5**), along with three genes mapping into isoflavone biosynthesis module gmx_M00941.

#### PI 567295 (moderately susceptible)

PI 567295 showed the highest number of DEGs at 5-DPI (570 DEGs, 83 upregulated, 487 downregulated), with enriched categories including transcriptional regulation and oxidative processes. However, the number of DEGs dropped significantly at 10-DPI (27 DEGs, 17 upregulated, 10 downregulated). KEGG pathway analysis revealed early enrichments in hormone signaling and secondary metabolite biosynthesis, with reduced pathway involvement at 10-DPI.

### Inter-line transcriptomic response analysis

Comparative analysis of DEGs across soybean lines at 5- and 10-DPI revealed distinct transcriptional responses SCN infection. The full list of inter-line DEGs is provided in **Supplementary Table 4**, with key results summarized below.

#### 5-DPI

**Figure 6:**
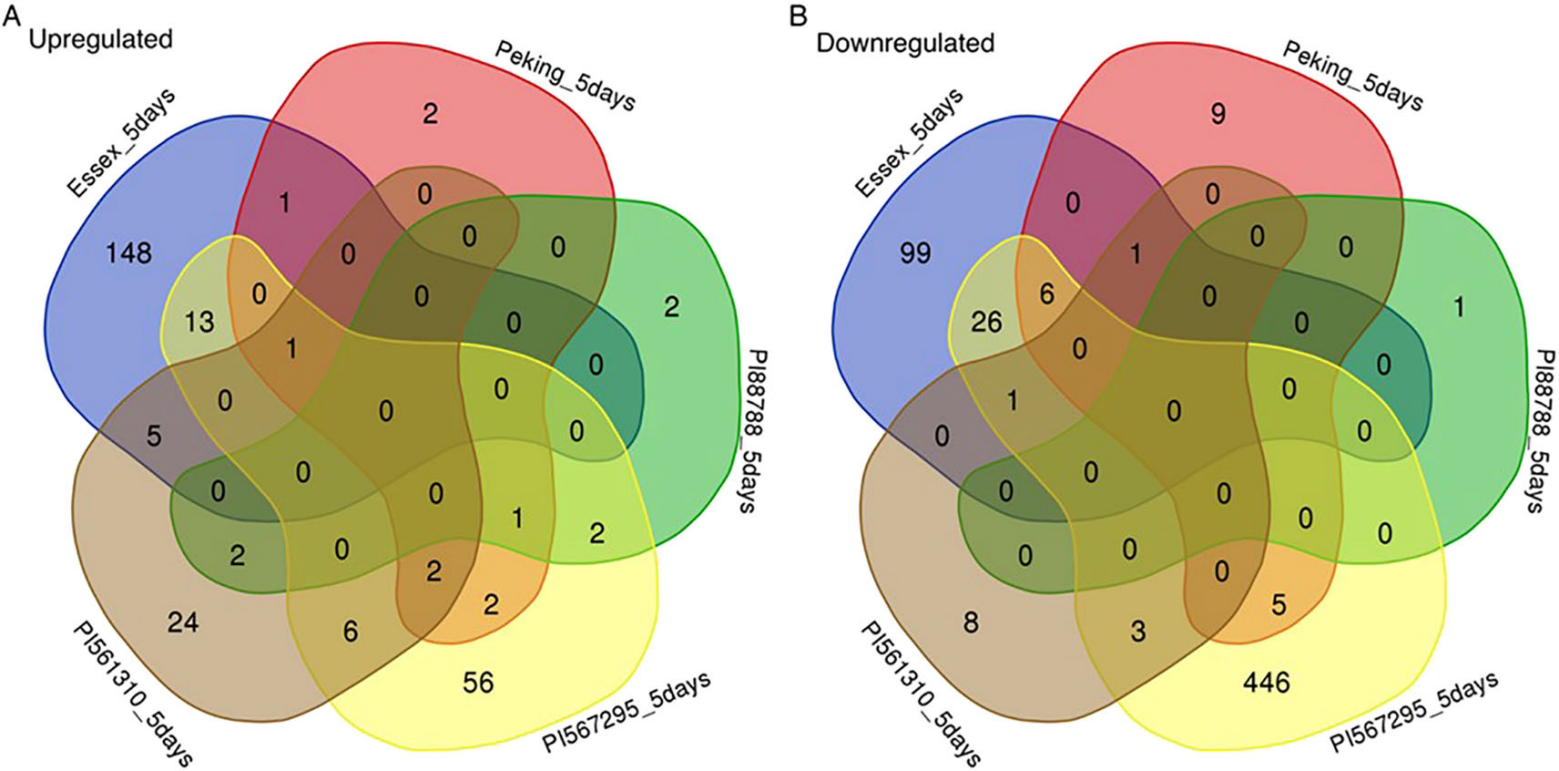
Five-way Venn diagrams depicting the overlaps of differentially expressed genes from each of the five soybean lines at 5-DPI, divided into **(A)** upregulated genes identified in infected compared to non-infected treatments, and **(B)** downregulated genes identified in infected compared to non-infected treatments. Non-overlapping areas represent genes identified as uniquely expressed within each line, whereas the intersections represent differentially expressed genes identified as distinctly shared by the representative lines. Essex is shown in blue, Peking is shown in red, PI 88788 is shown in green, PI 567295 is shown in yellow, and PI 561310 is shown in brown.

At 5-DPI, Essex displayed a unique response with 247 exclusive DEGs, including upregulated genes enriched as ethylene-responsive transcription factors and downregulated genes related to MYB transcription factors (**Figure 6**). PI 561310 showed a moderate response with 32 exclusive DEGs, characterized by upregulation of Cytochrome P450 family proteins, while PI 567295 had the largest exclusive response (502 DEGs), with significant downregulation of genes involved as heat shock proteins and glutathione S-transferases. Notably, some genes, such as Glyma.08G339600, were upregulated in PI 561310 but downregulated in Peking, highlighting contrasting regulatory patterns.

#### 10-DPI

**Figure 7:**
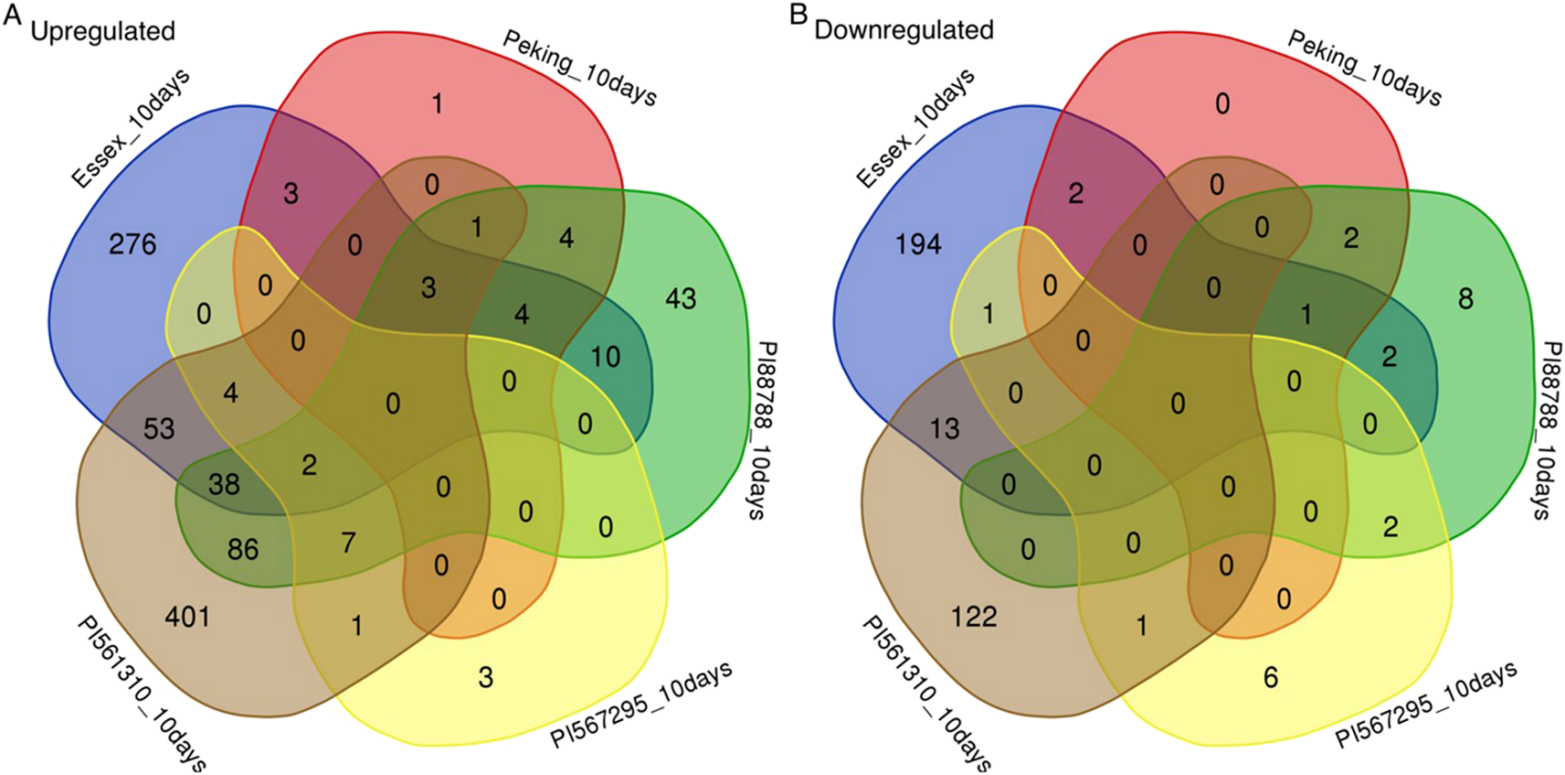
Five-way Venn diagrams depicting the overlaps of differentially expressed genes from each of the five soybean lines at 10-DPI, divided into **(A)** upregulated genes identified in infected compared to non-infected treatments, and **(B)** downregulated genes identified in infected compared to non-infected treatments. Non-overlapping areas represent genes identified as uniquely expressed within each line, whereas the intersections represent differentially expressed genes identified as distinctly shared by the representative lines. Essex is shown in blue, Peking is shown in red, PI 88788 is shown in green, PI 567295 is shown in yellow, and PI 561310 is shown in brown.

At 10-DPI, Essex again exhibited a strong response, with 470 exclusive DEGs, including upregulated genes enriched as response regulator proteins and downregulated genes from the WRKY transcription factor family (**Figure 7**). PI 561310 displayed the most substantial transcriptional activity with 523 exclusive DEGs, notably upregulated BON1-associated proteins, while PI 567295 showed minimal response, with only nine exclusive DEGs. Shared regulatory contrasts were observed, such as Glyma.13G094900, which was upregulated in Essex but downregulated in PI 567295.

This analysis underscores the variability in transcriptional responses to SCN infection among soybean lines, with Essex and PI 561310 showing the most pronounced exclusive DEG patterns and enriched pathways related to stress response and regulatory processes, such as cell wall modification (**Table 3**).

**Table 3:** DEGs mapped as peroxidases in the KEGG phenylpropanoid pathway that are observed shared between lines at the two time-points. Information includes identified locus tags/glyma IDs corresponding to Glycine max Williams 82 reference version 2.1, the line and time-point of the DEG and corresponding log_2_ fold change of the DEG, if observed as present.

Gene	Essex 5-DPI	Essex 10-DPI	Peking 5-DPI	Peking 10-DPI	PI 88788 5-DPI	PI 88788 10-DPI	PI 561310 5-DPI	PI 561310 10-DPI	PI 567295 5-DPI	PI 567295 10-DPI
Glyma.09G277900	NA	NA	3.66	NA	NA	1.97	3.17	5.03	3.61	2.53
Glyma.11G162100	2.73	NA	NA	NA	NA	4.57	NA	6.04	3.59	3.09
Glyma.02G233800	NA	NA	NA	NA	5.42	2.97	2.89	5.03	NA	NA
Glyma.06G145300	1.50	NA	NA	NA	NA	NA	NA	4.79	NA	2.38
Glyma.02G259300	NA	1.46	NA	NA	NA	NA	NA	3.46	1.78	NA
Glyma.14G201600	NA	NA	NA	NA	NA	3.32	NA	4.29	NA	NA
Glyma.09G022800	NA	NA	NA	NA	NA	NA	NA	5.53	2.68	NA
Glyma.09G277800	NA	NA	NA	NA	NA	NA	NA	3.72	1.39	NA
Glyma.11G058100	NA	-1.34	NA	NA	NA	NA	NA	-1.70	NA	NA
Glyma.18G055600	NA	NA	NA	NA	NA	5.40	NA	NA	NA	8.49

Color represents strength of the log_2_ fold change, with higher values more red on the spectrum, and lower values more green on the spectrum. Absence of DEG within a particular line and time-point denoted as NA.

### RT-qPCR validation of DEGs from RNA sequencing analysis

In order to verify the transcriptome sequencing data, we identified seven DEGs that were observed to be co-regulated in PI 561310 and PI 567295 (**Table 4**) with defense-related biological processes, and evaluated them across all lines by RT-qPCR. RT-qPCR expression analysis was deemed consistent with the results obtained from the differential expression data obtained from the RNA sequencing experiment (**Supplementary Figure 1**).

**Table 4:** DEGs identified as distinctly co-regulated in PI 561310 and PI 567295 at either time-points.

Gene name	Arabidopsis Tair10 annotation	Time point of differential expression	Direction of differential expression
Glyma.02G054200	Jasmonic acid carboxyl methyltransferase	5-DPI	Upregulated
Glyma.06G137000	2-oxoglutarate (2OG) and Fe(II)-dependent oxygenase superfamily protein	5-DPI	Upregulated
Glyma.06G145300	Peroxidase superfamily protein	10-DPI	Upregulated
Glyma.06G300400	MYB domain protein 74	5-DPI	Upregulated
Glyma.08G360700	SAUR-like auxin-responsive protein family	5-DPI	Downregulated
Glyma.09G274000	WRKY DNA-binding protein 70	5-DPI	Upregulated
Glyma.20G162300	Protein kinase superfamily protein	10-DPI	Downregulated

Information includes identified locus tags/glyma IDs corresponding to Glycine max Williams 82 reference version 2.1, time-point of co-expression identification and direction of differential expression observed in infected compared to non-infected samples.

## Discussion

### PCA and DEG profile analysis - temporal resistance responses

It is well-established that Peking and PI 88788 employ distinct resistance mechanisms against SCN infection, albeit from shared loci. Peking-type resistance is conferred by low-copy number *rhg1-a* in an epistatic interaction with *Rhg4*, that leads to a rapid hypersensitive response that results in syncytial collapse and the death of nematodes at the J2 stage, as early as 2-DPI (Klink et al., 2009; Mahalingam and Skorupska, 1996). This early activation likely explains the minimal number of DEGs observed in Peking at both 5- and 10-DPI, as the key defense responses may have already been initiated and resolved by these later time-points. On the other hand, PI 88788-type resistance is attributed to the high-copy number *rhg1-b*, resulting in a prolonged resistance reaction in which nematodes die at the J3 and J4 stages (Acedo et al., 1984; Klink et al., 2010). This is consistent with the observed DEG profile in PI 88788, in which the numbers of DEGs increase dramatically from just eight genes at 5-DPI to over 200 genes 10-DPI, corroborating that the defense mechanisms are being triggered later in this line. Importantly, the haplotypes conferring Peking-type and PI 88788-type resistances have been found to exhibit elevated and constitutive transcript levels even in the absence of SCN infection and as corroborated in our RNA sequencing data and other independent studies, the *rhg1* and *Rhg4* genes are not identified as differentially expressed in Peking and PI 88788 lines between non-infected and infected treatments (Chu et al., 2022; Cook et al., 2014, 2012; Lee et al., 2015; Torabi et al., 2023; Zhang et al., 2017)

At both 5-DPI and 10-DPI, both the infected and non-infected replicates form genotype specific clusters. Furthermore, PI 561310 and PI 267295 are observed distinctly separated from one another, as well as from PI 88788, Peking and Essex, indicative of differing patterns of gene expression. PI 561310 shows a substantial increase in DEGs from 5-DPI to 10-DPI, going from 53 to 732 genes, with the majority of differential gene expression being positive at both time-points. This substantial increase in gene regulation over time implies that PI 561310 may employ defense responses that progress and escalate as the infection progresses, peaking later in the infection cycle. In contrast, PI 567295 shows a marked decrease in DEGs from 5-DPI and 10-DPI, going from 570 to 27 genes, in which the majority of differential gene expression is negative at both time-points. Previous work with PI 567295 infected with SCN had reported that J2 nematodes were able to penetrate roots at 2-DPI and initiate syncytium formation, but by 4-DPI delayed nematode development at the J2 stage and evidence of degenerating syncytium around the head of J2 SCN was found (Tran, 2022). At 8-DPI, a clear necrosis of roots were observed, indicating that the plant had initiated a defense response and produced a necrotic layer to disconnect the syncytium from the vascular cylinder starving the females and arresting further development. The transcriptomic response paired with previous cytological observations suggests that the initial response of PI 567295 to SCN infection is substantial, but stabilizes by 10-DPI; consistent with a return to a homeostatic state.

### PI 561310 resistance via the phenylpropanoid pathway and its downstream influences

The phenylpropanoid pathway and its associated downstream pathways were found to be differentially regulated across lines in response to SCN infection to varying degrees, with the most pronounced regulation observed in PI 561310. The phenylpropanoid pathway is an important metabolism pathway in plants responsible for the biosynthesis of a variety of secondary metabolites including lignin, flavonoids and phenolic acids, from phenylalanine.

#### Lignin biosynthesis

The compounds generated by the phenylpropanoid pathway serve as precursors for the synthesis of lignin, an important structural component of cell walls and a modulator of defense responses. Modulation of the cell wall is a dynamic process initiated in response to cell wall damage, with lignin deposition occurring as a localized defense mechanism to limit pathogen spread by acting as a non-degradable mechanical barrier (Denness et al., 2011; Hamann, 2012; Moura et al., 2010; Naoumkina et al., 2010). Peroxidases catalyze the final step of lignin biosynthesis within the phenylpropanoid pathway. Various genes encoding for peroxidases (K00430, EC:1.11.1.7) were observed to be differently regulated under SCN infection. While no single peroxidase gene was consistent across all lines or times, there were notable differences regarding the concerted effect, including the coordination of the strength, direction and timing, of peroxidase regulation between the lines (**Table 3**). At 5-DPI, the observed upregulation of identified peroxidases across lines may indicate an initial attempt by the plants to reinforce their cell walls through lignin deposition, with distinct patterns emerging across the lines by 10-DPI. While Essex and Peking displayed limited or diminishing activity, PI 88788 and PI 561310 demonstrated increased peroxidase gene regulation, suggesting these lines employ cell wall modifications as part of their defense strategies. This temporal shift in pathway activation could be in response to the considerable number of genes upregulated upstream in the phenylpropanoid pathway, with the subsequent production of intermediates that are shunted into lignin biosynthesis to a more significant degree, as observed with the seven upregulated genes mapped into the KEGG module of monolignol biosynthesis (gmx_M00039). Overall, the lignin biosynthesis branch of the phenylpropanoid pathway was found differentially regulated across soybean lines under SCN infection, and as found in other studies, is further evidence of this pathway playing a fundamental role in the defense response soybean initiates against infection (Miraeiz et al., 2020; Torabi et al., 2023; Zhang et al., 2022).

#### Flavonoid biosynthesis

The recruitment of phenylalanine into the phenylpropanoid pathway can lead to the downstream biosynthesis of flavonoids, such as flavanones and isoflavones. Isoflavone biosynthesis is dependent on the production of two flavanone precursors: liquiritigenin and naringenin, to produce key isoflavone compounds such as glycitein, genistein and daidzein; which have also been found to potentially play a role in defense in soybean against SCN (Wang et al., 2020; Yang et al., 2024). Daidzein, whose production is catalyzed by 2-hydroxyisoflavanone dehydratase (K13258, EC:4.2.1.105), is then used to synthesize the phytoalexins glyceollins and coumestrol (Naoumkina et al., 2010). Phytoalexins are compounds that plants produce in response to attack by pathogens in an attempt to inhibit invasion, with glyceollins observed to exhibit anti-pathogenic activity and nematode-resistance (Huang and Barker, 1991; Lygin et al., 2010; Veech, 1982). The biosynthesis of pterocarpan glyceollin isomers (I, II or III) from daidzein requires at least 21 enzymes, including chalcone reductase (CHR), isoflavone synthase (IFS), isoflavone reductase (IFR), pterocarpan synthase (PTS) and CYP93A1 (Dixon and Steele, 1999; Nwachukwu et al., 2013; Xia et al., 2023). Despite the numerous enzyme intermediaries, Zhou et al. (2011) illustrated the importance of daidzein in the formation of phytoalexins and the role that the isoflavonoid biosynthesis pathway plays in the modulation of defense response, when they identified that a HopZ1 variant, a type III secreted effector from *Pseudomonas syringae*, interacts with and inhibits 2-hydroxyisoflavanone dehydratase and subsequently increases susceptibility to infection. Aliferis et al. (2014) strengthened this association when they found that soybean infected with *Rhizoctonia solani* resulted in an increase in daidzein, which in turn led to an increased accumulation of coumestrol and glyceollins. An important enzyme intermediary in the formation of glyceollins from daidzein is the cytochrome P_450_ CYP93A1. CYP93A1 has dihydroxy-pterocarpan 6a-hydroxylase activity and is so significantly involved in the synthesis of glyceollin that its induction was observed to result in a direct increase in glyceollin accumulation in elicitor treated soybean cell cultures (Kinzler et al., 2016). The genes responsible for catalyzing the final committed step of glyceollin biosynthesis have been unknown until recently, when Khatri et al. (2024) identified and characterized *GmGS11A* and *GmGS11B*, encoded by Glyma.11G062500 and Glyma.11G062600 respectively, which catalyze the conversion of glyceollidin to glyceollin I, and *GmGS13A*, encoded by Glyma.13G285300, which converts glyceocarpin to glyceollin III. In addition to the upregulation of genes involved in the biosynthesis of flavonoids observed in PI 561310, several genes of the daidzein and glyceollin biosynthesis pathways were upregulated; which leads us to believe that glyceollin synthesis is occurring and exerting an effect in PI 561310 in response to SCN infection. We propose that the enhanced production of these compounds positively impact defense response against SCN HG type 0.

Resistant soybean lines infected with SCN were not only found to have increased levels of isoflavone and glyceollin regulation, but also elevated PAL transcription levels; a response that is absent in susceptible lines (Mazarei et al., 2011; Yang et al., 2024; C. Zhang et al., 2017). Phenylalanine ammonia-lyase (PAL, K10775, EC: 4.3.1.24) is a critical enzyme that incorporates metabolites from the shikimate pathway into the phenylpropanoid metabolism pathway by catalyzing the first step of deaminating L-phenylalanine, producing trans-cinnamic acid and ammonia (R00697) (Edens et al., 1995; Yang et al., 2024). Serving as the entry point, PAL acts as a critical regulator between primary and secondary metabolism and directly influences multiple downstream pathways by regulating the availability of essential intermediates produced by the phenylpropanoid pathway, whose biosynthesis and accumulation have been associated with increased defense against infection with various pathogens (Edens et al., 1995; Erb and Kliebenstein, 2020). Supporting the role of PAL facilitating defense against SCN, Yang et al. (2024) found that the over-expression of PAL genes in a susceptible soybean line such as Williams 82 resulted in increased flavonoid and isoflavone content, reduction of the total number of nematodes in roots compared with controls, and inhibition of nematode development. Importantly, the specific *PAL* gene that was over-expressed impacted these variables, with overexpression of *GmPAL2.1*, *GmPAL2.2*, *GmPAL2.3*, *GmPAL2.4* or *GmPAL3.1* resulting in more substantial reductions in the total number of infested nematodes in the root system than over-expression of *GmPAL1.1*, *GmPAL1.2*, or *GmPAL1.3*. We observed the upregulation of Glyma.03G181600 and Glyma.03G181700, which encode for *GmPAL1.3* and *GmPAL1.2* respectively, in the moderately susceptible PI 561310 at 10-DPI, with Glyma.03G181700 found downregulated in the susceptible Essex at the same time-point. Taken together, this evidence accounts for the higher resistance exhibited by PI 561310 compared to the susceptible Essex.

#### Salicylic acid biosynthesis

In addition to its impact on flavonoid and lignin biosynthesis, PAL plays a direct role in the formation of salicylic acid (SA) in soybean (Zhang et al., 2017). Salicylic acid is a crucial signaling molecule in the activation of systemic acquired resistance (SAR), with roles in pathogen-associated molecular pattern-triggered immunity (PTI) and effector-triggered immunity (ETI) in plants. During SAR, pathogen-infected plants sites accumulate SA, which is then methylated by salicylic acid methyl transferase-like protein (SABATH2/SAMT1) using SAM as the methyl donor to form methyl salicylate (MeSA) (Lin et al., 2013). MeSA then travels to distal tissues and is subsequently reconverted into SA by SABP2 for SAR in leaves (Gong et al., 2023). Chorismic acid, which is derived from the shikimate pathway, is an important precursor that can be converted to SA through both the PAL- and the isochorismate synthase (ICS)-catalyzed pathways (Lefevere et al., 2020). In the PAL-catalyzed pathway, trans-cinnamic acid is oxidized to benzoic acid by Abnormal Inflorescence Meristem 1 (AIM1), which is then hydroxylated via a hypothetical BA-2-hydroxylase (BA2H) to produce SA (Peng et al., 2021). Unlike other plant species, both the PAL- and ICS-catalyzed pathways contribute significantly to the production of SA in soybean. Shine et al. (2016) established the importance of PAL in the production of SA in soybean when their silencing of PAL genes resulted in significantly reduced levels of SA and inhibited pathogen resistance. Furthermore, Zhang et al. (2017) found that PAL genes regulate SA levels following infection of soybean with *Phytophthora sojae* infection. *SAMT1* is a gene known to confer resistance to SCN in soybean. Not only has the upregulation of *SAMT1* been observed in a number of studies in resistant soybean lines infected with SCN, it has been proven to play a role in soybean defense response against SCN when its overexpression in susceptible backgrounds significantly reduced the SCN burden against multiple races, in both lab and field tests (Lin et al., 2016, 2013; Mazarei et al., 2011; Zhang et al., 2017). Our results found upregulation of Glyma.02G054200, which encodes for SAMT1, in both PI 561310 and PI 567295 at 5-DPI, but also PI 561310, PI 88788 and Essex at 10-DPI. At 5-DPI, PI 561310 presented with a substantially higher log_2_ fold change for *SAMT1* compared to PI 567295, resulting in a ˜7.5x stronger response in PI 561310. Importantly, while *SAMT1* was found upregulated in Essex at 10-DPI, it does so with a substantially lower log_2_ fold change compared to PI 88788 and PI 561310, resulting in Essex having a ˜26x and ˜5x weaker response than PI 561310 and PI 88788, respectively. These results illustrate that the strength of the response is an important variable in SCN resistance. Interestingly, the corresponding Arabidopsis TAIR10 annotation of *SAMT1* is S-adenosyl-L-methionine:jasmonic acid carboxyl methyltransferase (JMT); a component of the JA pathway which catalyzes the formation of methyl jasmonate from jasmonic acid, with its expression induced in response to wounding or methyl jasmonate treatment. This discrepancy highlights the need for species-specific studies.

### PI 567295 resistance is unique and divergent

Soybean cyst nematode resistance in soybean is a complex polygenic trait and typically cannot be attributed to a single molecular player or mechanism operating autonomously. Canonical resistance mechanisms function via the synergistic and concerted contributions of multiple molecular components and signaling pathways that enable the plant to mount a robust and effective defense response against pathogens. A subset of genes previously identified to confer resistance against SCN was found in PI 567295, and predominantly at 5-DPI, supporting previous cytological findings (Tran, 2022). At 5-DPI, PI 567295 was observed to have upregulated *SAMT1*, *GmGS13A*, peroxidase genes, WRKY-family genes and MYB-family genes. WRKY and MYB are two families of transcription factors that regulate signal transduction and have roles in PTI and ETI (Han et al., 2022). WRKY family members possess a highly conserved WRKY domain and act in the regulation of downstream target genes via binding of cis-acting elements in the promoter regions, whereas MYB members possess a highly conserved MYB DNA-binding domain at the N-terminus and a variable activation domain at the C-terminus, and have a wide range of regulatory functions (Du et al., 2012; Yang et al., 2017). Of the differentially expressed WRKY genes identified, Glyma.13G267600 was not only found upregulated in PI 567295 at 5-DPI, but was also found in Peking and PI 561310 at the same timepoint. Zhang et al. (2017) found that Glyma.13G267600 was the most strongly induced *WRKY70* gene following infection with SCN HG type 2.5.7, increased by six-fold, in the resistant wild soybean line S54. Discordantly, most of the differentially expressed MYB genes identified in PI 567295 were downregulated, and when compared to the 31 differentially regulated MYB genes presented by Zhang et al. (2017), only Glyma.10G191000 and Glyma.06G299900 were identified and similarly regulated. In fact, when comparing the list of identified DEGs from PI 567295 with those reported in studies focused on similar objectives using different soybean lines or SCN populations, we consistently found minimal overlap. This deviation could indicate that PI 567295 employs a distinct molecular strategy in response to infection with SCN HG type 0.

Moreover, PI 567295 exhibits a distinct expression profile in response to SCN infection, both compared to the other infected lines studied, but also within the scope of the available literature. Soybean cyst nematode infection is known to induce global transcriptomic changes in soybean, with the observed trend of an increased ratio of upregulated to downregulated SCN responsive genes (Hewezi et al., 2018; Rambani et al., 2020a, 2020b). In contrast, in response to infection with SCN type 0, PI 567295 was observed to have initiated a significant transcriptional response at 5-DPI predominantly composed of downregulated genes. The only other instance of this pattern of response was found when the resistant wild soybean ecotype NRS100 was infected with SCN HG type 2.5.7 (race 5) (Kofsky et al., 2021). Although, the comparison of PI 567295 at 5-DPI with HG type 0 and NRS100 at 6-DPI with HG type 2.5.7 shows a greater quantity of expressed genes in NRS100 than in PI 567295, and unfortunately, as with other DEG analyses, gene expression similarities are limited. Ultimately, while a minor subset of DEGs in PI 567295 following SCN infection have been previously confirmed or correlated with roles in SCN resistance, the majority of the genes currently lack supporting evidence. Additionally, PI 567295 exhibited extensive differential gene regulation at 5-DPI, with 570 DEGs, that reduced drastically, to under 30 genes, by 10-DPI; a significant reversal indicative of a dynamic response.

Considering these findings, we propose that the mechanism modulating the resistant phenotype in PI 567295 may be more heavily based on epigenetic regulation. Epigenetics refers to heritable changes in gene expression that does not alter the underlying genetic sequence. Biochemical modifications, microRNAs and long non-coding RNAs modulate gene expression at the transcriptional and post-transcriptional levels on a genome-wide scale. Epigenetic regulation is a complex and dynamic mechanism that allows for rapid and reversible responses, driving phenotypic plasticity (Cadavid et al., 2023; Pikaard and Mittelsten Scheid, 2014; Yang et al., 2018). When looking at dynamic, genome-wide changes in gene expression, particularly negative regulation, microRNA (miRNA)-mediated post-transcriptional gene silencing comes to mind. Mature miRNAs are small, around ˜20 nucleotides long, non-coding RNAs that cause post-transcriptional gene silencing via cleavage of target transcripts possessing complementary sequences (Hewezi et al., 2016). Indeed, post-transcriptional gene silencing via the action of miRNAs, alongside the modulation of epigenetic marks, have been implicated in soybean-nematode interactions (Han et al., 2022, 2017; Hewezi et al., 2018; Rambani et al., 2020a, 2020b). While further research of this hypothesis falls beyond the scope of this study, the results gathered so far highlights the reaction of PI 567295 to SCN infection with HG type 0 as highly atypical and particularly intriguing. PI 567295 appears to present as a novel source of resistance unlike anything currently presented in the literature and our results provide direction for its further elucidation and characterization.

## Conclusion

In this study, we evaluated the transcriptional responses of five soybean lines presenting with various degrees of resistance to Soybean Cyst Nematode (SCN) HG type 0 infection. Essex was susceptible to infection, whereas Peking and PI 88788 were resistant to infection through known *rhg*-based resistance mechanisms. Two lines, PI 561310 and PI 567295, were previously determined to not possess the resistant alleles at *rhg1* or *Rhg4* and exhibited partial resistance identified respectively as moderately resistant and moderately susceptible. We employed RNA sequencing on the root tissues collected from each line under non-infected and infected conditions at 5 and 10 days post-infection to capture the gene expression changes resulting from the dynamic interplay between infection progression, defense response and stress management. We concluded that the two uncharacterized PI lines employ distinct resistance mechanisms based on PCA plot segregation and differences in the transcriptomic profiles. Following comparative transcriptomic analysis and functional annotation, enrichment and pathway analyses, we discovered that the resistance mechanism employed by PI 561310 appears to be driven by the expression of *PAL*, the gateway enzyme into the phenylpropanoid pathway. The upregulation of *PAL* isoforms *PAL1.2* and *PAL1.3* following SCN infection in PI 561310 subsequently and positively regulated downstream lignin, flavonoid and salicylic acid biosynthesis pathways, leading to an apparent increase in lignified cell walls, flavonones and isoflavanoids such as phytoalexins, and salicylic acid. While unable to elucidate the resistance mechanisms employed by PI 567295 following SCN infection, we observed a unique and divergent transcriptomic response. At 5-DPI, PI 567295 was observed to have initiated a significant response, whereby genes were predominantly downregulated, as oppose to canonically upregulated, that was then stabilized by 10-DPI. We hypothesize that the resistance mechanism mounted by PI 567295 is influenced heavily by epigenetic regulation and our results provide direction for further work. This study presents important findings into novel SCN resistance as we navigate the landscape of evolving SCN parasitism.

## Data Availability

The datasets presented in this study can be found in online repositories. The names of the repository/repositories and accession number(s) can be found in the article/**Supplementary Material**.
